# Clawed forelimbs allow northern seals to eat like their ancient ancestors

**DOI:** 10.1098/rsos.172393

**Published:** 2018-04-18

**Authors:** David P. Hocking, Felix G. Marx, Renae Sattler, Robert N. Harris, Tahlia I. Pollock, Karina J. Sorrell, Erich M. G. Fitzgerald, Matthew R. McCurry, Alistair R. Evans

**Affiliations:** 1 School of Biological Sciences, Monash University, Melbourne, Victoria, Australia; 2 Geosciences, Museums Victoria, Melbourne, Victoria, Australia; 3 Directorate of Earth and History of Life, Royal Belgian Institute of Natural Sciences, Brussels, Belgium; 4 Alaska SeaLife Center, Seward, AK, USA; 5 Sea Mammal Research Unit, Scottish Oceans Institute, University of St Andrews, St Andrews, UK; 6 National Museum of Natural History, Smithsonian Institution, Washington, DC, USA; 7 Department of Life Sciences, Natural History Museum, London, UK; 8 Australian Museum Research Institute, Sydney, New South Wales, Australia; 9 PANGEA Research Centre, School of Biological, Earth and Environmental Sciences, University of New South Wales, Sydney, New South Wales, Australia

**Keywords:** feeding behaviour, marine mammals, forelimb anatomy, claws, pinnipeds, evolution

## Abstract

Streamlined flippers are often considered the defining feature of seals and sea lions, whose very name ‘pinniped’ comes from the Latin *pinna* and *pedis*, meaning ‘fin-footed’. Yet not all pinniped limbs are alike. Whereas otariids (fur seals and sea lions) possess stiff streamlined forelimb flippers, phocine seals (northern true seals) have retained a webbed yet mobile paw bearing sharp claws. Here, we show that captive and wild phocines routinely use these claws to secure prey during processing, enabling seals to tear large fish by stretching them between their teeth and forelimbs. ‘Hold and tear’ processing relies on the primitive forelimb anatomy displayed by phocines, which is also found in the early fossil pinniped *Enaliarctos*. Phocine forelimb anatomy and behaviour therefore provide a glimpse into how the earliest seals likely fed, and indicate what behaviours may have assisted pinnipeds along their journey from terrestrial to aquatic feeding.

## 1. Introduction

The hunt for new food resources was one of the key factors driving the repeated re-invasion of aquatic ecosystems by whales, seals and other secondarily aquatic mammals [1]. But switching from feeding in air to feeding entirely underwater presents significant challenges requiring major adaptations to both anatomy and behaviour [2].

Air-breathing mammals need to hold their breath when diving to feed underwater, taking care not to drown while capturing prey in their mouths and processing it into pieces small enough to swallow. This can be challenging, because while prey on land can easily be held against the ground for processing using sharp cutting teeth, aquatic prey floats freely in the water column [3]. Swallowing can itself also become an issue, as aquatic mammals must separate prey from surrounding seawater or bear the high physiological cost of ingesting it along with their prey. Meanwhile, all of this is further complicated by the need to dramatically modify their body form to increase streamlining and to adapt their limbs for generating propulsion when swimming [4]. In response to these challenges, marine mammals have adopted a myriad of aquatic feeding strategies, involving behaviours which are often so highly specialized that they would be impossible to use on land [5,6].

While making this switch from terrestrial to aquatic feeding, marine mammals must have gone through an intermediate stage, where they were still using more-or-less ancestral anatomy and behaviour to capture and consume aquatic prey. Echoes of this transition can be seen in living semi-aquatic mammals like river otters, which typically capture prey underwater using their teeth and forelimbs, before bringing it on to land for processing [7]. Land-based processing helps these mammals avoid some of the main challenges of fully aquatic foraging, as seawater simply drains off their prey and it allows them to breathe freely during lengthy bouts of prey processing [5,6]. Semi-aquatic feeding, where prey is captured underwater but processed in air, therefore plausibly assists in smoothing the evolutionary transition between terrestrial and fully aquatic feeding.

Like otters, modern pinnipeds are descended from terrestrial carnivoran ancestors, and hence may have displayed a similar suite of semi-aquatic feeding behaviours prior to the evolution of their more specialized aquatic strategies. But while otters are able to use their dexterous forelimbs to capture and secure prey, the transformation of forelimbs into flippers for swimming makes doing so more difficult for many modern pinnipeds [3]. Fur seals and sea lions (otariids), in particular, have specialized flippers which make ineffective tools for gripping prey during processing [8,9]. By contrast, northern true seals (phocines) have retained a webbed paw bearing sharp claws, closely resembling that of living terrestrial carnivorans like wolverines or bears [10,11]. Terrestrial mammals employ their clawed forelimbs for a variety of tasks, which raises the question of how modern seals make use of this relatively primitive anatomy. Are clawed forelimbs really just a relic of their distant past, or do they still play an important role in phocine ecology?

To explore these questions, we first compared phocine forelimbs to those of early fossil seals and modern terrestrial carnivorans, to see how similar their current morphology is to that of their presumed ancestors. We then made close observations of phocine behaviour by performing both wild field observations and captive animal experiments. Our results suggest that phocines have retained not only primitive morphology, but also a suite of behaviours similar to those likely used by the earliest seals, providing important insights into how behaviour and anatomy interplay as species cross the boundary between terrestrial and aquatic existence.

## 2. Material and methods

### 2.1. Anatomical observations

To determine how forelimb anatomy influences feeding behaviour in phocines, we examined two captive harbour seals (*Phoca vitulina*) and a spotted seal (*Phoca largha*) at the Alaska SeaLife Center in Seward, AK, USA. To compare phocine skeletal anatomy with that of other pinnipeds and terrestrial carnivorans, we examined and photographed museum specimens in the mammalogy collections at Museums Victoria (NMV, Australia), the Smithsonian Institution National Museum of Natural History (USNM, USA) and the American Museum of Natural History (AMNH, USA). To compare phocines with the earliest known pinnipeds, we examined a near-complete skeleton (USNM 374272) referred to *Enaliarctos mealsi* [12].

### 2.2. Wild observations

We observed the feeding behaviour of harbour seals and grey seals (*Halichoerus grypus*) as part of routine studies investigating the interactions between seals and wild salmon fisheries in Scotland. The Sea Mammal Research Unit has been conducting these studies since 2005 to fulfil contractual commitments to the Scottish Government. Over this time, observers have regularly recorded wild seals predating large salmonid fish. The most recent study focused on the River Dee, Aberdeenshire, Scotland, where one of the authors (Robert Harris) made observations between April 2016 and March 2017 [13]. Whenever possible, feeding events were photographed using a Canon 600 mm f4 lens and 1.4 tele-converter with 7D mark 2 camera body (Canon Inc., Tokyo, Japan).

### 2.3. Captive feeding trials

To gain a closer view of phocine feeding behaviour than is possible in the wild, we carried out captive feeding experiments with the harbour and spotted seals at the Alaska SeaLife Center. The study subjects included a captive-born male (PV84) and female (PV11) harbour seal, born in 1984 and 2011, respectively; and one female wild-born spotted seal, PL16, admitted into the rehabilitation facility in May 2016 at an estimated age of 1 year (table 1). The harbour seals were housed in outdoor public display pools and thus exposed to a natural light cycle and environmental conditions, whereas the spotted seal was kept in an indoor pool with a simulated light cycle.

**Table 1.** The species, sex, age at sampling (age), weight (WT), standard length (SL), curvilinear length (CL) and axial girth (AG) for the three northern phocine seals used in our captive feeding trials.

animal ID	species	sex	age (years)	WT (kg)	SL (cm)	CL (cm)	AG (cm)
PV84	*Phoca vitulina*	M	30	73.5	147	156	107.5
PV11	*Phoca vitulina*	F	5	57	154.6	133.4	94
PL16	*Phoca largha*	F	1	33.8	106	116.5	85

We performed feeding trials between June and August 2016. Seals were fasted overnight prior to each trial, and then offered a single large thawed pink salmon (*Oncorhynchus gorbuscha;* mean ± s.d. weight = 1.33 ± 0.31 kg, fork length = 47.57 ± 3.32 cm, body depth = 10.28 ± 1.11 cm). Feeding behaviours were recorded until the seals had either consumed or discarded the fish. Behaviours were filmed from both above and below the water using GoPro HERO 3 movie cameras (GoPro, San Mateo, CA) mounted on PVC pipes held by the observers to track the animal's movements.

Footage from multiple cameras was edited into a single movie file and then imported into Behaviour Observation Research Interactive Software [14] to tally the number of bouts for each foraging behaviour per animal/trial. Behaviours were coded based on the ethogram in table 2. Whenever a behaviour could not be clearly coded, e.g. because the animals had moved out of sight, the bout in question was discarded from the analysis. As a result, the counts presented here represent minimum estimates.

**Table 2.** Ethogram and operational definitions for prey processing methods used by phocine seals when feeding on large prey. Data were recorded as bouts of behaviour, where the animal could perform multiple prey-processing actions (e.g. multiple shakes) as part of a single bout with pauses or different behaviours in between bouts. Hence this analysis recorded the number of bouts of each behaviour that were performed, rather than how many individual processing actions were performed within each bout.

foraging behaviour	description and operational definition
gulping to swallow large prey	prey was swallowed by jerking the head backwards while simultaneously biting down on prey. This appeared to force the prey backwards into the oesophagus.
hold and tear at surface	holding and stretching prey between the teeth and forelimbs to create the tensional load that tears the prey item. This load was applied by pulling the item away from the mouth with forelimbs while simultaneously arching the head back using the neck. When performed at the surface either the head or both the head and forelimbs were positioned above the surface of the water at the start of the bout. Each bout was recorded from when the seal first holds the prey item with both its teeth and forelimbs. The bout ended if the seal dropped the prey item or if the prey item tore.
hold and tear underwater	same as hold and tear feeding at surface, but performed with both the head and forelimbs positioned underwater. This could be performed completely underwater (near the bottom of the pool) or near the surface where the animal was still facing down in the water so only their back breached the surface. Each bout was recorded from when the seal first holds the prey item with both its teeth and forelimbs, and ended if the seal dropped the prey item or if it tore.
shaking at surface	holding prey in teeth and flicking from side to side so that its own inertia causes it to tear. A bout of shake feeding commenced with the first flick and ended if the seal dropped the prey item or if the prey item broke. Hence, one bout of shaking could involve multiple shakes or flicks of the prey item.
shaking underwater	holding prey in teeth and shaking from side to side underwater. Pulling against drag in the water, rather than the inertia of the prey item, created the tensional load that caused the prey item to tear. The bout ended if the seal dropped the prey item or if the prey item tore.
securing with paw on land	prey was held against the ground on land beneath the palm of the forelimb and/or with the digits flexed so that the claws dug into the prey item. The seal then pulled away from the secured prey item by arching its neck. This applied tension as the prey item was stretched between the teeth and forelimb. A new bout was recorded when the prey was held down beneath the flipper and gripped with the teeth. The bout ended if the seal dropped the prey item or the prey item tore.

## 3. Results

### 3.1. Anatomical observations

**Figure 1.**
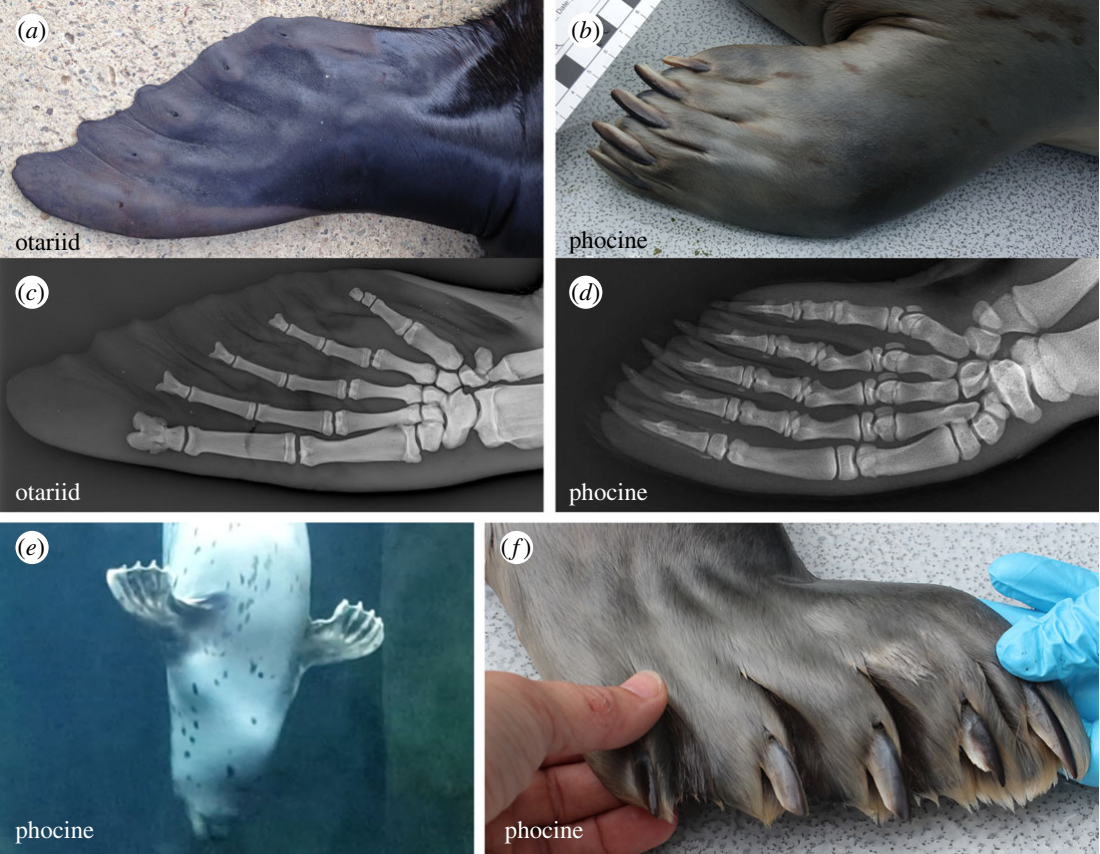
Rather than bearing typical flippers like those of otariids (*a*), phocine seals have paw-like forelimbs with distinct digits and robust claws (*b*). (*a,b*) External forelimb anatomy for otariids (male Australian sea lion *Neophoca cinerea*) and phocines (female harbour seal *Phoca vitulina*—PV11). (*c,d*) Skeletal anatomy of the forelimb in otariids (long-nosed fur seal *Arctocephalus forsteri*—mirrored) and phocines (juvenile harbour seal). The radiograph image in (*d*) was taken under the Alaska SeaLife Center's NOAA/NMFS Stranding Agreement. (*e*) Harbour seal (PV11) showing webbing between spread digits during swimming. (*f*) Harbour seal (PV11) showing distinct and mobile digits with strong claws.

The anatomy of the phocine paw is less derived than that of the otariid flipper: otariid forelimb digits are integrated to form a stiff, wing-like flipper, whereas phocine digits are distinct and mobile (figure 1*a,b*). The extent to which phocines can flex their digits is clearly indicated by the presence of saddle-shaped trochleated phalangeal articulations, which contrasts with the flattened articulations found in otariids (figure 1*c,d*). Phocines use their forelimbs as paddles and rudders while swimming [15], and when the digits are spread underwater the webbing stretched between them is clearly visible (figure 1*e*). Webbing is less obvious when the paw is flexed for use during terrestrial locomotion or feeding (figure 1*f*). Phocines also retain strong claws which are supported internally by long and robust ungual processes (figure 1*b,d*). These claws are so long that they protrude well beyond the webbing, breaking the streamline of the paddle-like paw during swimming (figure 1*e*).

**Figure 2.**
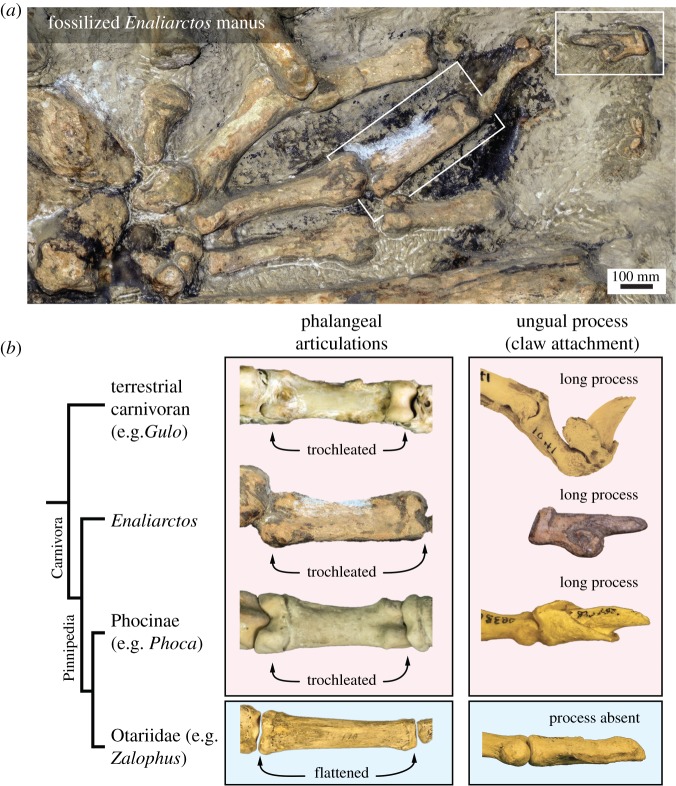
Evolution of digit morphology in pinnipeds. (*a*) Forelimb manus morphology in one of the earliest fossil seals, *Enaliarctos mealsi* (USNM 374272). Boxes indicate elements discussed in (*b*). (*b*) Phylogeny showing the relative positions of a generalized terrestrial carnivoran (wolverine *Gulo gulo*, NMV C30416), an early pinniped (*Enaliarctos*), a phocine seal (harbour seal *Phoca vitulina*, NMV C27683) and an otariid (California sea lion *Zalophus californianus,* USNM 14410). Trochleated articulations (in dorsal view) and robust claws (in lateral view) are present in all but otariids, indicating that phocines inherited this morphology from their terrestrial ancestors, via the earliest seals. *Enaliarctos* ungual mirrored in (*b*).

Like phocines, the forelimbs of archaic pinniped *Enaliarctos mealsi* also feature trochleated phalangeal articulations, suggesting that it too could curl its digits (figure 2*a*). Likewise, the preserved ungual phalanges bear a long bony process indicating the presence of a strong claw. These features are typical of terrestrial carnivorans (e.g. the wolverine *Gulo gulo*), and so likely represent the ancestral condition inherited by the earliest pinnipeds from their land-living ancestors (figure 2*b*).

### 3.2. Wild observations

**Figure 3.**
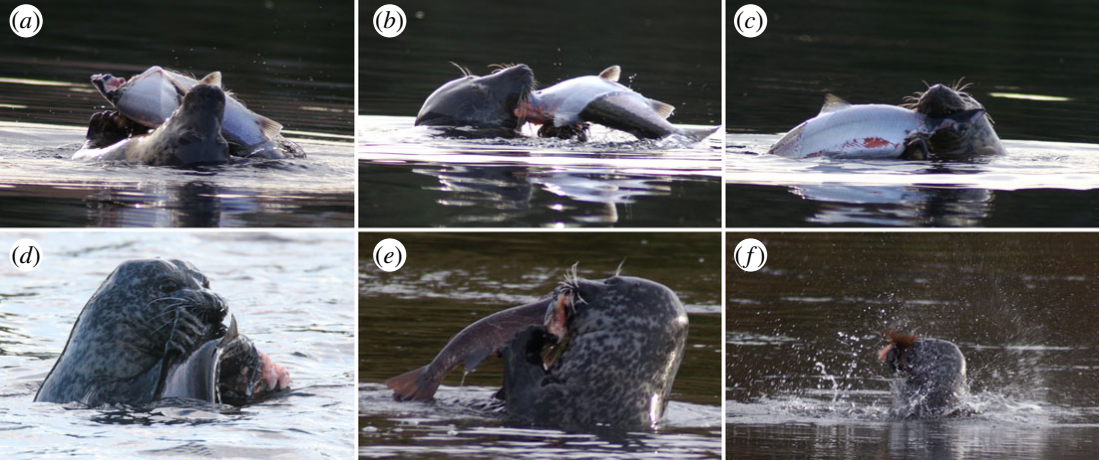
Prey processing in wild phocine seals. (*a–c*) Wild grey seal (*Halichoerus grypus*) peeling the skin off a large Atlantic salmon (*Salmo salar*) before tearing it into pieces small enough to swallow using both teeth and forelimbs. (*d,e*) Wild harbour seals (*Phoca vitulina*) using their clawed paws to securely grip prey. (*f*) Wild harbour seal shaking or thrashing prey to process it into smaller pieces for swallowing.

We conducted 670 h of field observations, resulting in the documentation of 123 wild feeding events where either harbour or grey seals were seen feeding on a range of large salmonid fish, including Atlantic salmon *Salmo salar* [13]. Smaller fish (relative to the size of the seal) were generally swallowed whole, while larger fish were first dismembered at the water's surface (figure 3). Prey processing often began by either removing the head or by attacking the fish's soft belly. The seals used their teeth to hold the lower jaw or gill coverings, before gripping the body using their clawed forelimbs and pushing away at the prey item, causing it to tear. As the seals processed the fish, they often succeeded in pulling strips of skin away from the trunk, exposing the flesh underneath (figure 3*a–c*). While most processing observed involved using the teeth and forelimbs to tear prey, seals were occasionally seen shaking or thrashing prey at the surface (figure 3*f*). This processing behaviour appeared to be used more often by harbour seals, the smaller of the two species, but was generally uncommon in either species. However, it was not possible to quantify the relative frequency of shaking or thrashing due to the challenges of closely observing animals at a distance in the field.

### 3.3. Captive feeding trials

In total, we recorded 20 informative feeding trials (PV84 *n* = 10; PV11 *n* = 7; PL16 *n* = 3). The seals initially captured prey by biting, and wherever possible, swallowed their catch whole without processing. This was especially true of the male harbour seal (PV84), which routinely used a ‘gulping’ action with jerky neck/head movements accompanied by biting, seemingly to force prey backwards into the oesophagus (electronic supplementary material, movie S1).

**Figure 4.**
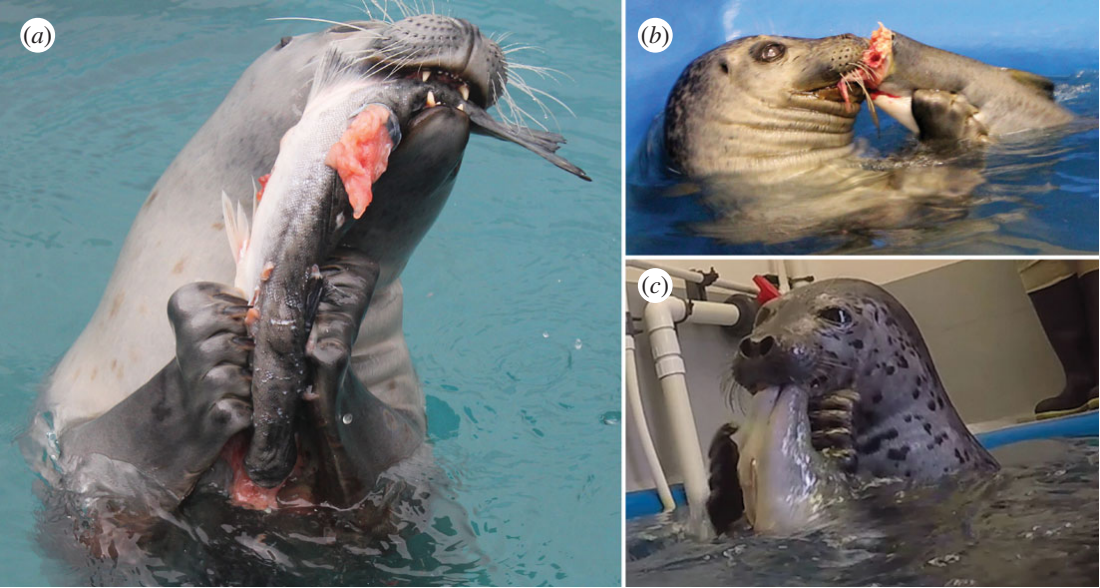
Phocine seals curl their digits so that the strong claws can be used to effectively grip prey during processing. (*a*) Female harbour seal (*Phoca vitulina*, PV11) using both forelimbs together to grip prey. The long claws can clearly be seen piercing the skin. This processing attempt was unsuccessful because the seal was trying to tear the whole fish, rather than focussing on pulling off a smaller piece. (*b*) Male harbour seal (PV84) showing a more typical processing posture: the teeth grip a small piece of flesh, while the forelimbs pull the main bulk of the prey item away from the mouth. (*c*) Spotted seal (*Phoca largha*, PL16) holding prey between its teeth and forelimbs just before commencing a processing attempt. See electronic supplementary material, movie S1, for the footage of these feeding events.

**Figure 5.**
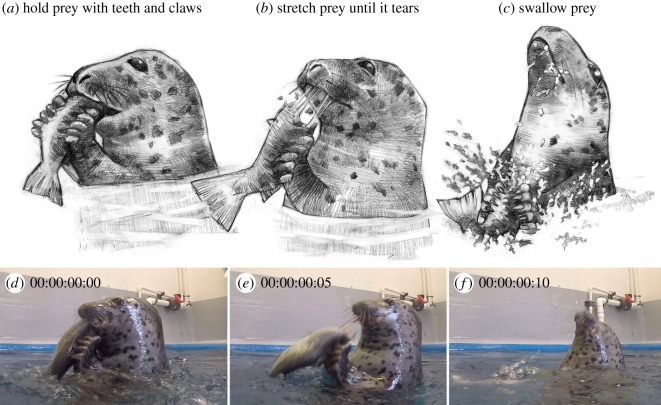
Phocine seals use ‘hold and tear’ prey processing to break large prey into pieces small enough to swallow. (*a*) First, the seal holds prey using both its anterior teeth and clawed paws. (*b*) The seal then arches its head back using its flexible neck, while simultaneously pulling down with its forelimbs. This stretches the prey, causing it to tear. (*c*) Once a small piece has been torn free, it can then be swallowed. By performing this behaviour in air, seals avoid the difficulties of separating prey from seawater and holding their breath during processing. (*d–f*) Captive spotted seal (*Phoca largha*) performing hold and tear processing while eating a large pink salmon (*Oncorhynchus gorbuscha*). Time displayed as hours : minutes : seconds : frames. See electronic supplementary material, movie S1, for the footage of this feeding event.

**Figure 6.**
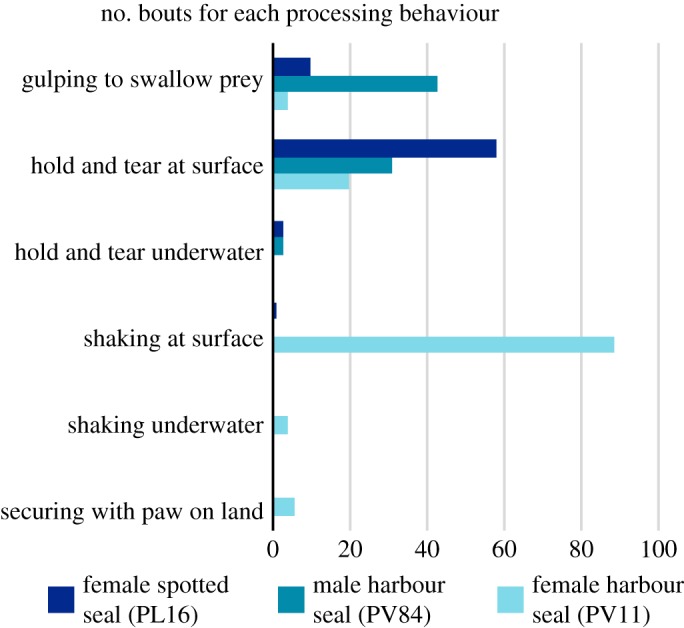
Number of behavioural bouts performed for each type of prey processing observed during these trials. See electronic supplementary material, tables S1–S3, for the breakdown of each feeding trial.

Like the wild seals, processing typically involved tearing prey between the teeth and forelimbs. Unlike their wild counterparts, however, the captive individuals did not routinely peel back the skin of their prey, possibly because of differences in prey consistency between fresh and frozen/thawed salmon. While tearing prey, the seals firmly held the fish between their canines and/or incisors, while simultaneously gripping it with their paws. They curled their digits so that the claws dug into the prey, sometimes piercing the skin (figure 4). They then arched their head backwards while pulling down with the forelimbs, stretching the prey item and causing it to tear (figure 5; electronic supplementary material, movie S1). ‘Hold and tear’ processing was typically performed at the surface, but PV84 and PL16 occasionally also performed this behaviour underwater (figure 6; electronic supplementary material, movie S1).

Processing performance appeared to depend on individual levels of experience. For example, the spotted seal, PL16, began processing large fish effectively from the first trial, suggesting that it acquired the necessary skills during its first year of life in the wild (electronic supplementary material, movie S1). By contrast, the two captive-born harbour seals had more difficulty. The male, PV84, had been fed large prey in the past, but not recently prior to these trials. He always initially attempted to swallow the fish whole, and only moved on to ‘hold and tear’ processing if unsuccessful (electronic supplementary material, movie S1). The female, PV11, which had never been fed large prey, frequently failed at her attempts to tear it apart. As a result, she resorted to thrashing the fish into pieces, often while hauled out on the shore (electronic supplementary material, movie S1). On land, she also tried to use her paws to grasp the prey, but struggled because of her posture (lying on her belly rather than floating vertically) and the shortness of her forelimbs (electronic supplementary material, movie S1). Both of the captive-born seals became more skilled as they gained experience through the trials.

As well as in their behaviour, the seals also differed in the time it took to consume prey. The spotted seal (PL16) took 12.3 ± 0.42 min (mean ± s.e.) to process its prey, whereas the two harbour seals were considerably quicker (PV84 = 3.48 ± 1.07 min, PV16 = 6.55 ± 1.58 min). This difference may be related to the age and size of the individuals, with the harbour seals being larger (electronic supplementary material, table S1) and hence presumably able to swallow bigger pieces of prey.

## 4. Discussion

Our results demonstrate that wild phocine seals routinely use their forelimbs to assist with processing large prey. This is made possible by their relatively primitive forelimb morphology, including flexible digits and strong claws. Semi-aquatic feeding behaviours, where prey is captured underwater before being brought to the surface for processing, were likely important when pinnipeds first began the transition from feeding on land to feeding in water [5,6]. Unlike more specialized aquatic feeding strategies common in living pinnipeds (e.g. suction feeding), semi-aquatic behaviours would have allowed early seals to successfully capture and consume aquatic prey while using much of their existing repertoire of behaviours.

Modern terrestrial and semi-aquatic carnivorans (e.g. wolverines and otters) also routinely use their forelimbs to secure prey while it is processed using the teeth [7,11]. One important distinction, though, is that terrestrial mammals typically chew on their prey with sharp carnassial teeth, a feature which modern pinnipeds lack. Instead of cutting their food using bladed cheek teeth, phocines use their anterior teeth to grip prey, while simultaneously stretching and tearing it with their forelimbs. Early seals like *Enaliarctos* retained the occluding dentition of their terrestrial ancestors, and thus plausibly could have used their sharp cheek teeth to cut prey, rather than relying solely on tearing like their modern descendants [12,16–18]. By manipulating prey using its dexterous forelimbs, *Enaliarctos* would have been able to control which teeth it used during processing, allowing it to either cut or tear its prey. An interesting parallel of this is found in sea otters (*Enhydra lutris*) which use crushing molars to crack open hard-shelled prey, while soft prey is torn between their anterior teeth and forelimbs [19,20].

By capturing prey underwater, but processing it in air, early seals like *Enaliarctos* would have avoided some of the major pitfalls of fully aquatic feeding. In particular, semi-aquatic behaviours would have made it easy to drain seawater from prey before swallowing, and would have allowed them to breathe freely while processing their catch [5,6]. Both features may have helped the earliest seals to begin exploiting aquatic prey: rather than having to change their feeding style completely, they were able to redeploy much of their existing anatomy and behaviour to the new task of feeding in water.

In the past, there have been suggestions that phocine forelimb morphology may not be truly primitive, but a rare example of ‘evolutionary regression’ [21,22]. However, recent molecular and morphological phylogenies indicate that it is at least equally parsimonious that phocines inherited this anatomy from their ancestors [23,24]. Amson and de Muizon [23], in particular, suggested that phocines more likely retained, rather than reinvented, their paws, a point with which we firmly agree.

The retention of primitive forelimbs in modern phocines enables them to perform a number of tasks that could be thought of as ‘terrestrial’ rather than ‘aquatic’. For example, despite being well known for their undulating mode of terrestrial locomotion, phocines in fact extensively use their forelimbs to haul themselves forward when traversing uneven surfaces, especially when young [10,25]. Even more surprisingly, some phocines routinely use their forelimbs for digging. For example, ringed seals (*Pusa hispida*) use their strong claws to excavate lairs in ice during breeding seasons [26], while wild harbour seals have been observed to dig into seafloor sediments to flush buried fish out of hiding [27]. Finally, grey seals occasionally use their forelimbs to assist with feeding on land, and have been seen using them to hold prey firmly against the ground while cannibalizing young seals [28,29].

The use of forelimbs for ‘hold and tear’ processing may provide phocines with an advantage by enabling more efficient processing of large prey, which can be more profitable than targeting smaller fish [27]. The use of forelimbs to secure and tear large prey may also be essential in allowing grey seals to predate upon other marine mammals, such as harbour porpoises *Phocoena phocoena* [30]. However, ‘hold and tear’ processing is not the only way to handle large prey. Fur seals and sea lions with flipper-like forelimbs instead usually grip prey in their teeth before shaking or thrashing their food at the water's surface [8,9]. This behaviour is also used by some southern phocids (monachines) including leopard seals (*Hydrurga leptonyx*) and Weddell seals (*Leptonychotes weddellii*) [31,32]. Interestingly, monachines appear to be converging on otariid-like forelimb morphology, which may explain why they no longer use their reduced claws to secure prey during processing [33].

In addition to otariids and monachines, this trend towards increasing aquatic specialization of the forelimb is also present in walruses (*Odobenus rosmarus*), the sole surviving member of the pinniped family Odobenidae. Like otariids, walruses have reduced claws and cartilage extending each of their digits [34]. Walruses use their broad flippers to push water, both during swimming and, somewhat surprisingly, to fan sediment-free water in front of their faces to help them see the seafloor while searching for buried shellfish [35,36]. Species with flipper-like forelimbs may still be able to hold prey by squeezing it between their flat palms (e.g. [9,37]), but this is likely a less effective way to secure prey compared with the phocine method of gripping and piercing prey using strong claws.

One advantage of the ‘hold and tear’ processing behaviours used by phocines over the ‘shaking’ behaviours favoured by otariids and monachines is that the former presents fewer opportunities for other predators to steal their catch, a behaviour known as kleptoparasitism. While shaking involves tossing prey around at the surface [8], hold and tear enables the predator to keep a closer hold on its meal. Interestingly, during our wild observations, seals were often seen to carry fish in their jaws away from the site of capture to a more secluded area for processing using the forelimbs, possibly as another way to avoid unwanted competition.

One of the reasons that these results are so surprising is that phocid seals are often considered to be more aquatically adapted than otariids. This perception is due, in part, to the extreme adaptation of the phocid hind limb for aquatic locomotion, which reduces their ability to move on land [10,25]. By contrast, otariids are still quite agile when moving rapidly over rocky shorelines. Phocids are also generally considered to be better divers than otariids, as exemplified by Weddell seals *Leptonychotes weddellii* and elephant seals *Mirounga* sp. [38]. In reality, however, all pinnipeds display a complex mixture of characteristics, including some that can be thought of as primitive. Just as otariids resemble their terrestrial ancestors in their fur, ears and agile hind limbs, so do phocids in the structure and use of their bear-like paws. Nevertheless, both phocids and otariids are effective marine predators that play important roles in modern ocean ecosystems.

The transition from feeding on land to feeding completely underwater at first seems like a daunting task, especially given the extreme specializations found in many modern marine mammals. Yet our results show that the earliest seals could effectively feed in water while still relying on the anatomy and behaviour of their terrestrial ancestors. Once they had made this first step into the water, it would have become easier to dive in further, building on these semi-aquatic behaviours to become the true marine mammals we know them as today.

## Supplementary Material

Hocking_tables_ESM.pdf

## Data accessibility

Additional data are available as the electronic supplementary material.
