# Supplementary material for: Clawed forelimbs allow northern seals to eat like their ancient ancestors
Source: R Soc Open Sci. 2018 Apr 18;5(4):172393. doi: 10.1098/rsos.172393 (PMC5936949; doi:10.1098/rsos.172393)
Supplement: Hocking_tables_ESM.pdf [file rsos172393supp1.pdf]

Supplementary Materials

Clawed forelimbs allow northern seals to eat like their ancient ancestors

David P. Hocking[1,2]*, Felix G. Marx[1-3], Renae Sattler[4], Robert N. Harris[5], Tahlia I. Pollock[1], Karina J. Sorrell[1], Erich M. G. Fitzgerald[2,6,7], Matthew R. McCurry[8,9] and Alistair R. Evans[1,2]

1. School of Biological Sciences, Monash University, Melbourne, Victoria, Australia.
2. Geosciences, Museums Victoria, Melbourne, Victoria, Australia.
3. Directorate of Earth and History of Life, Royal Belgian Institute of Natural Sciences, Brussels, Belgium.
4. Alaska SeaLife Center, Seward, Alaska, United States.
5. Sea Mammal Research Unit, Scottish Oceans Institute, University of St Andrews, St Andrews, United Kingdom.
6. National Museum of Natural History, Smithsonian Institution, Washington, D. C., United States.
7. Department of Life Sciences, Natural History Museum, London, United Kingdom.
8. Australian Museum Research Institute, Sydney, New South Wales, Australia.
9. PANGEA Research Centre, School of Biological, Earth and Environmental Sciences, University of New South Wales, Sydney, New South Wales, Australia.

*Author for correspondence: david@dphocking.com

Table S1. Number of behavioural bouts performed for each type of prey processing observed during these trials for the female harbour seal *Phoca vitulina* (PV11).

**Feeding behaviour:**	**Trial 1**	**Trial 2**	**Trial 3**	**Trial 4**	**Trial 5**	**Trial 6**	**Trial 7**
Hold and tear at surface	0	0	0	5	7	1	7
Hold and tear underwater	0	0	0	0	0	0	0
Shaking at surface	1	13	10	25	16	6	18
Shaking underwater	0	0	0	0	0	0	0
Securing with paw on land	0	1	1	1	3	0	0
Gulping to swallow prey	0	0	0	3	1	0	0
Feeding event duration (min)	1.92	4.42	6.68	14.83	6.90	3.50	7.62

Table S2. Number of behavioural bouts performed for each type of prey processing observed during these trials for the male harbour seal *Phoca vitulina* (PV84)

**Feeding behaviour:**	**Trial 1**	**Trial 2**	**Trial 3**	**Trial 4**	**Trial 5**	**Trial 6**	**Trial 7**	**Trial 8**	**Trial 9**	**Trial 10**
Hold and tear at surface	1	1	1	0	4	10	0	2	1	11
Hold and tear underwater	0	0	0	0	0	3	0	0	0	0
Shaking at surface	0	0	0	0	0	0	0	0	0	0
Shaking underwater	0	0	0	0	0	0	0	0	0	0
Securing with paw on land	0	0	0	0		0	0	0	0	0
Gulping to swallow prey	4	4	7	5	8	4	3	2	2	4
Feeding event duration (min)	1.85	3.90	2.27	1.78	10.57	6.78	0.87	2.37	0.90	11.23

Table S3. Number of behavioural bouts performed for each type of prey processing observed during these trials for the female spotted seal *Phoca largha* (PL16)

**Feeding behaviour:**	**Trial 1**	**Trial 2**	**Trial 3**
Hold and tear at surface	22	25	13
Hold and tear underwater	2	0	1
Shaking at surface	1	0	0
Shaking underwater	0	0	0
Securing with paw on land	0	0	0
Gulping to swallow prey	6	4	0
Feeding event duration (min)	12.98	11.53	12.38
